# Screening and exploration of neoadjuvant “de-escalation” therapy for early breast cancer

**DOI:** 10.3389/fphar.2025.1574665

**Published:** 2025-03-25

**Authors:** Nana Zhang, Ming Shan, Zhenfeng Huang, Fei Gao, Bingqi Xu, Wenli Kang, Jian Zhang, Li Song, Jun Liu, Jiawei Zhang, Mingyang Liu, Haitao Jiang, Xinhang Liu, Zibo Shen, Peng Zhang, Abiyasi Nanding, Guoqiang Zhang

**Affiliations:** ^1^ Department of Breast Surgery, Harbin Medical University Cancer Hospital, Harbin, Heilongjiang, China; ^2^ Department of Anorectum, Heilongjiang General Hospital of Daqing Oil Field, Daqing, Heilongjiang, China; ^3^ Department of Oncology, Beidahuang Group General Hospital, Harbin, Heilongjiang, China; ^4^ Department of Thyroid and Breast Surgery, The Third Affiliated Hospital of Shenzhen University, Shenzhen, Guangdong, China; ^5^ Department of Oncology, JiaMuSi Tumor Hospital, JiaMuSi, Heilongjiang, China; ^6^ Department of Breast Surgery, Dalian Municipal Friendship Hospital, Dalian, Liaoning, China; ^7^ Department of Oncology, WangKui County People’s hospital, WangKui, Heilongjiang, China; ^8^ Department of Oncology, Hailun People’s Hospital, Suihua, Heilongjiang, China; ^9^ Department of Pharmacy, First Affiliated Hospital, Heilongjiang University of Chinese Medicine, Harbin, China; ^10^ Biomedical and Life Science Faculty, King’s College London, London, United Kingdom; ^11^ Faculty of Economics and Management, Baotou Teachers’ College, Baotou, Inner Mongolia Autonomous Region, China; ^12^ Department of Pathology, Harbin Medical University Cancer Hospital, Harbin, Heilongjiang, China

**Keywords:** breast cancer, neoadjuvant chemotherapy, pathological complete response, “de-escalation” therapy, prognosis

## Abstract

**Background:**

Neoadjuvant therapy for breast cancer improves the prognosis of high-risk patients. However, whether pathological completed response (pCR) can be used as a surrogate endpoint for de-escalation therapy in patients who are relatively sensitive to treatment remains to be elucidated.

**Methods:**

We retrospectively reviewed 143 breast cancer patients, with clinical stage (cStage) II–IIIA who received neoadjuvant chemotherapy and achieved pCR in a short time (within 16 weeks) from 2012 to 2022. The prognosis of patients was analysed using the Kaplan-Meier method, Cox proportional hazards regression models to identify independent clinicopathologic factors affecting prognosis.

**Results:**

The median follow-up period was 47 months, the overall 4-year disease-free survival (DFS) and overall survival (OS) were 95.3% and 96.9%, respectively, in 143 patients with pCR after neoadjuvant chemotherapy. The 4-year DFS between the postoperative adjuvant chemotherapy and no adjuvant chemotherapy groups was 76.4% and 95.2%, with a significant statistical difference between both groups (*P* < 0.05). For HER2-positive (HER2+) and Triple negative breast cancer (TNBC), the addition of targeted therapy or platinum-based drugs had no impact on prognosis. Univariate and multivariate analyses of prognosis showed that only postoperative adjuvant chemotherapy significantly affected prognosis.

**Conclusion:**

Patients with operable cStage II–IIIA breast cancer who achieved pCR after a short period of neoadjuvant chemotherapy have a satisfactory prognosis and may be suitable for chemotherapy “de-escalation.” This approach is also a dominant application of neoadjuvant “tailoring therapy.”

## 1 Introduction

Neoadjuvant therapy has significantly transformed the treatment paradigm for early-stage breast cancer. By replacing the traditional model of adjuvant chemotherapy with neoadjuvant chemotherapy guided by pathological complete response (pCR) as a surrogate endpoint, this approach has demonstrated the potential to enhance treatment efficacy and improve prognosis, particularly in high-risk subtypes such as human epidermal growth factor receptor 2-positive (HER2+) and triple-negative breast cancer (TNBC) (Masuda et al., 2017; von Minckwitz et al., 2019). Concurrently, the field of neoadjuvant therapy is actively exploring de-escalation strategies, with pCR as a primary endpoint, to identify treatment-sensitive populations. These strategies aim to improve patient tolerance, alleviate financial burdens, enhance quality of life, and reduce the toxic side effects associated with chemotherapy (Spring et al., 2022).

Current research hotspots in this area primarily focus on two key directions. First, with the support of dual-target therapy, efforts are being made to reduce the side effects and long-term toxicity of chemotherapy by minimizing the use of anthracyclines, thereby improving tolerability in patients, particularly those with HER2+ breast cancer (van Ramshorst et al., 2018). Second, researchers are investigating the identification of novel biomarkers to screen for patients who are highly sensitive to targeted therapies. This approach aims to reduce chemotherapy intensity while maintaining or enhancing therapeutic efficacy through targeted therapy (Werutsky and Rosa, 2020; Hurvitz et al., 2018). These advancements underscore the shift toward personalized treatment strategies that balance efficacy with reduced toxicity, ultimately improving outcomes for breast cancer patients.

The use of pathological complete response (pCR) as a primary endpoint in breast cancer treatment raises questions about its accuracy in reflecting long-term survival benefits. While studies like the CTNeoBC pooled analysis show favorable prognoses for pCR patients (Cortazar et al., 2014; von Minckwitz et al., 2012), the Phase 3 NOAH trial revealed that HER2+ patients achieving pCR after high-intensity neoadjuvant chemotherapy had a 5-year EFS of 87% and OS of 91%, whereas those without targeted therapy had significantly worse outcomes (EFS: 55%, OS: 71%) (Gianni et al., 2010). Retrospective studies suggest adjuvant therapy post-pCR improves prognosis (Huang et al., 2020; Berruti et al., 2014), indicating that treatment regimens and chemotherapy intensity influence outcomes even in pCR patients. Prognostic systems like Neo-Bioscore and RCB highlight variability among pCR patients, with Neo-Bioscore showing DSS ranging from 71% to 99% (Mittendorf et al., 2016) and RCB-0 patients exhibiting differing prognoses based on Neo-Bioscore (Laas et al., 2021). Our prior studies identified factors such as pretreatment lymph node metastasis and staging affecting pCR prognosis (Asaoka et al., 2019; van Mackelenbergh et al., 2023; Abdelsattar et al., 2016; Huang et al., 2021), emphasizing the need to align neoadjuvant studies with traditional adjuvant regimens to preserve survival benefits.

Recent research, however, demonstrates that non-locally advanced, treatment-sensitive patients achieving pCR with low-intensity chemotherapy and targeted therapies can have excellent outcomes. The KRISTINE study found similar 3-year IDFS in pCR patients receiving T-DM1+P or TCH+P (96.7% vs 97.5%) (Hurvitz et al., 2019), while retrospective analyses confirm that effective drug-supported pCR does not compromise prognosis, regardless of adjuvant chemotherapy (Huang et al., 2021; Spring et al., 2020; Yee et al., 2020). Trials like CompassHER2, I-SPY2, GeparSixto, and NeoSphere (von Minckwitz et al., 2014; O'Sullivan et al., 2021; Park et al., 2016; Gianni et al., 2012) support short-course neoadjuvant chemotherapy for HER2+ and TNBC, enabling expedited surgery for responsive patients or those with poor chemotherapy tolerance. Postoperative decisions on chemotherapy continuation are tailored to patient conditions, potentially deviating from traditional adjuvant cycles.

This retrospective study analysed treatment-sensitive, operable stage II–IIIA breast cancer patients achieving pCR after short-course neoadjuvant chemotherapy (≤16 weeks) without completing standard regimens. By integrating these findings with prior analyses, we identified pCR patients who may not require adjuvant therapy, laying the groundwork for individualized neoadjuvant de-escalation (Tailor Therapy) and improved patient selection, moving beyond traditional adjuvant approaches.

## 2 Materials and methods

### 2.1 Patient population

This study retrospectively analysed patients with TNBC and Her2+ breast cancer who received NAC and achieved pCR at the Harbin Medical University Cancer Hospital from March 2012 to July 2022 (Table S1). The main inclusion criteria were as follows: (1) pathologically diagnosed invasive ductal carcinoma of the breast; (2) breast cancer stage II–IIIA (AJCC 7th edition); (3) receipt of NAC (Her2 + able to receive targeted therapy); (4) surgical treatment following NAC; and (5) surgical pathology confirming the patient achieved pCR. The main exclusion criteria were as follows: (1) no surgical treatment after neoadjuvant chemotherapy; (2) incomplete pathological immunohistochemical information; (3) patients with neoadjuvant chemotherapy for more than 16 weeks; (4) systemic metastasis; and (5) incomplete follow-up data. The demographic characteristics studied were as follows: disease stage and subtype, treatment regimen, recurrence, and survival of patients. The data were extracted from electronic medical records, and the cut-off date for follow-up was January 2023. All procedures performed in this study were in accordance with the ethical standards of institutional and/or national research councils, as well as the 2023 Declaration of Helsinki and its subsequent amendments or comparable ethical standards. The retrospective study design was approved by the Ethics Committee of Harbin Medical University Cancer Hospital, and written informed consent was obtained from each patient prior to participation.

### 2.2 Pathologic assessment

Pathologists evaluated biopsy specimens obtained from core needle biopsies for each patient to assess hormone receptor (HR), HER2, and Ki67 status. Immunohistochemical staining (IHC) was performed in the Department of Pathology at Harbin Medical University Cancer Hospital to determine these markers. HR positivity was defined as estrogen receptor (ER) ≥ 1% or progesterone receptor (PR) ≥ 1%. HER2 positivity was determined according to the American Society of Clinical Oncology/College of American Pathologists (ASCO/CAP) guidelines: HER2 3+ by IHC or HER2 2+ with confirmation of HER2 amplification via fluorescence *in situ* hybridization (FISH). FISH criteria included a HER2/chromosome enumeration probe 17 (CEP17) ratio ≥2.0 with a mean HER2 copy number ≥4.0 signals per cell, or a HER2/CEP17 ratio <2.0 with a mean HER2 copy number ≥6.0 signals per cell. Based on HR and HER2 status, patients were classified into intrinsic subtypes: HER2-positive, triple-negative, or luminal (HR+/HER2-).

Ki67 expression was evaluated using the MIB-1 antibody (Dako, Glostrup, Denmark) on tumor tissue. Ki67 scoring was performed using a global scoring method, with the percentage of tumor cells showing nuclear staining calculated among all cancer cells. Pathological complete response (pCR) was defined as the absence of residual invasive carcinoma in both the breast and lymph nodes (ypT0/isypN0). Lymph node response to neoadjuvant therapy was assessed based on the presence of significant fibrosis, degeneration, necrosis, histiocyte accumulation, calcification, cholesterol crystal formation, multinucleated giant cell reaction, or other treatment-related changes. All pathological evaluations were independently conducted by two experienced pathologists. In cases of disagreement, a consensus was reached through consultation to ensure consistent and accurate results.

### 2.3 Neoadjuvant chemotherapy and surgery

The neoadjuvant chemotherapy regimens used for this study were as follows: TAC (docetaxel 75 mg/m^2^, epirubicin 75 mg/m^2^, and cyclophosphamide 500 mg/m^2^ every 3 weeks); AC-T (epirubicin 90 mg/m^2^ and cyclophosphamide 600 mg/m^2^ every 3 weeks, followed by docetaxel 100 mg/m^2^ every 3 weeks); TCb (docetaxel 75 mg/m^2^, carboplatin AUC 6 mg/mL/min, every 3 weeks); TC (docetaxel 75 mg/m^2^ and cyclophosphamide 600 mg/m^2^ every 3 weeks); AC (epirubicin 75 mg/m^2^ and cyclophosphamide 600 mg/m^2^ every 3 weeks); and AT (docetaxel 75 mg/m2 and epirubicin 75 mg/m^2^ every 3 weeks). In HER2-positive patients, chemotherapy combined with targeted therapy (every 3 weeks), including single-target trastuzumab (intravenously at 6 mg/kg on day 1 of each 21-day cycle after a loading dose of 8 mg/kg) or dual-target trastuzumab combined with pertuzumab (trastuzumab, intravenously at 6 mg/kg on day 1 of each 21-day cycle after a loading dose of 8 mg/kg; and pertuzumab, intravenously at 420 mg on day 1 of each 21-day cycle after a loading dose of 840 mg) is recommended.

Following completion of neoadjuvant chemotherapy, patients undergo radical breast surgery, axillary dissection, or sentinel lymph node dissection. Decisions to undergo breast-conserving surgery were made by consensus between the patient and surgeon. Axillary lymph node dissection was used after completion of neoadjuvant chemotherapy for all patients who developed metastatic disease in the axillary region at the time of the diagnosis of a core needle biopsy before chemotherapy. If there was no lymph node involvement, lymph node dissection or sentinel lymph node biopsy was performed according to the patient’s wishes. Postoperative radiotherapy was performed if the patient underwent breast-conserving surgery. Fifty Gy in 25 fractions was prescribed for these patients. HER2-positive patients continued to receive the targeted therapy every 3 weeks for 1 year postoperatively, according to the targeted application regimen during neoadjuvant therapy. Postmenopausal patients with HR+ endocrine therapy were treated with aromatase inhibitors for more than 5 years, while premenopausal patients were treated with tamoxifen.

### 2.4 Statistical analysis

Univariate and multivariate analyses were performed using Cox proportional hazards regression models to identify high-risk factors associated with survival outcomes. Results were reported as hazard ratios (HRs) with corresponding 95% confidence intervals (CIs). All statistical tests were two-sided, and *P* < 0.05 was considered statistically significant (Zhai et al., 2024; Xiao et al., 2019; Xiao et al., 2020; Zhang et al., 2024). Data analysis was conducted using SPSS version 22 software (IBM Corp., Armonk, NY, USA).

## 3 Results

### 3.1 Clinicopathological features and treatment outcomes in pCR patients

**FIGURE 1:**
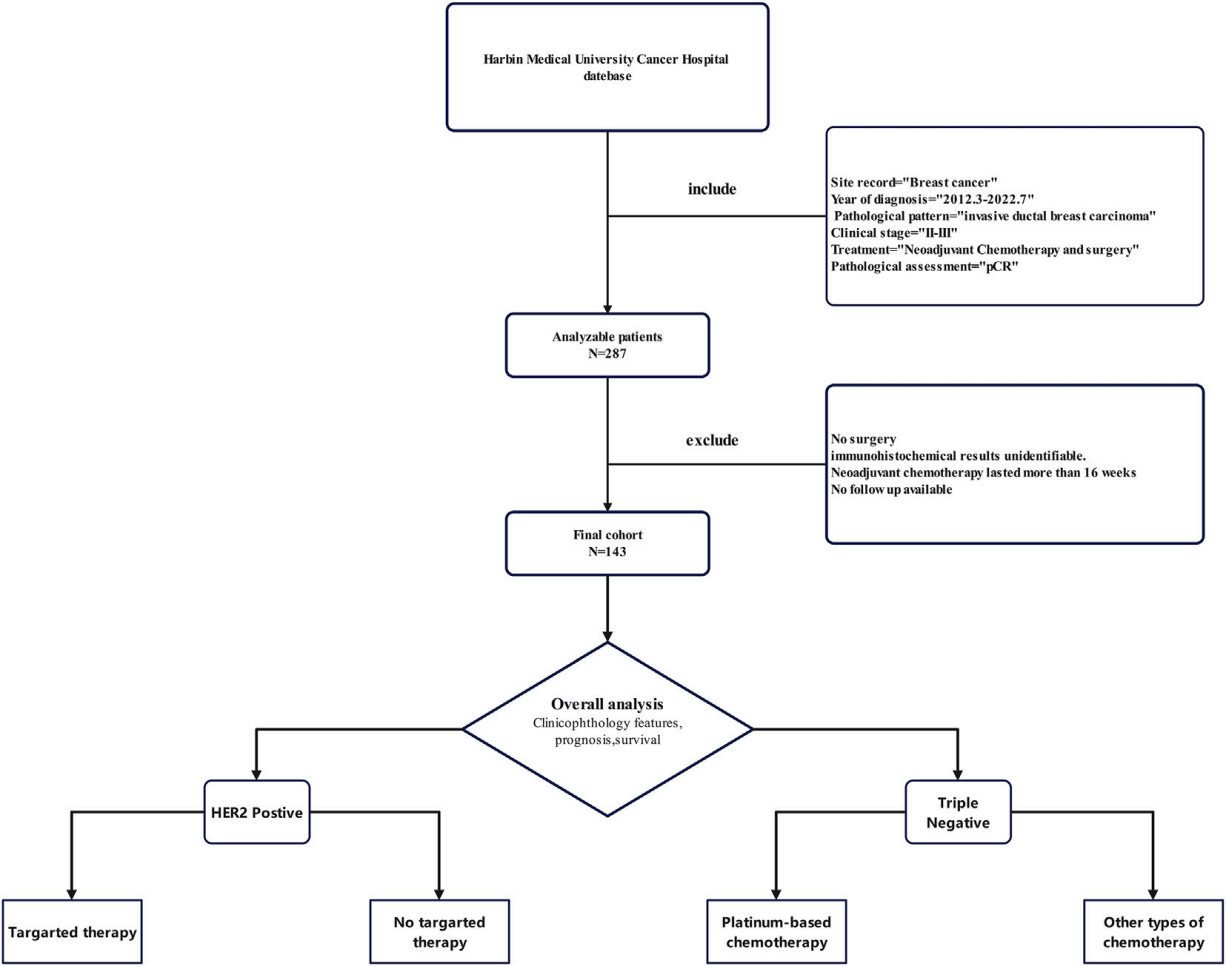
CONSORT Flow Diagram of patients who achieved pCR available for analysis.

A total of 143 patients with breast cancer with clinical stage II-IIIA and neoadjuvant chemotherapy were included (Figure 1). All patients underwent neoadjuvant chemotherapy and surgery. Pathology confirmed there was no residual invasive breast cancer in the primary tumour or axillary lymph nodes postoperatively. The clinicopathological characteristics of patients are shown in Table 1. The mean age of patients was 52 years, with 43.4% and 56.6% of patients being premenopausal and postmenopausal, respectively. Among different tumour stages, stages cT2 (67.8%) and cN1 (58.1%) comprised the largest proportion of the cohort. Among patients with different subtypes, TNBC and HER2-positive breast cancer patients comprised the majority, with rates of 42.7% and 51.7%, respectively. Only 8 (5.6%) hormone receptor-positive patients achieved pCR after a short course of neoadjuvant chemotherapy. The corresponding regimens were mainly anthracyclines combined with taxanes (52.4%), followed by taxanes combined with platinum drugs (30.8%). Most patients (68.5%) were treated with a 3-week regimen, while 45 (31.5%) were treated with a dose-dense regimen (single-week treatment). Preoperative chemotherapy was administered for less than 12 weeks in 125 patients (87.4%) and for 12–16 weeks in 18 patients (12.6%). Most patients (89.5%) did not receive subsequent adjuvant chemotherapy after surgery to achieve pCR. Among HER2+ patients, 26 patients (35.1%) received targeted therapy preoperatively, of whom 16 (21.6%) were treated with single-target therapy, while 10 (13.5%) were dual-target. Forty-eight patients (64.9%) also achieved pCR preoperatively, without receiving any targeted therapy. Postoperative pathologic evaluation revealed that there were 25 (17.5%) patients with residual ductal carcinoma *in situ* and 118 (82.5%) without any residual cancer in the breast. However, pathological evaluation of the dissected axillary lymph nodes revealed that a post-treatment pathological response was observed in lymph nodes in 65 (45.5%) patients.

**TABLE 1:** Characteristics of Neoadjuvant therapy patients with PCR.

Characteristic	pCR (*n* = 143)
Age at diagnosis(years)	
≤50	55 (38.5%)
>50	88 (61.5%)
Menstrual status	
Premenopausal	62 (43.4%)
Postmenopausal	81 (56.6%)
Clinical T classification	
cT1	31 (21.7%)
cT2	97 (67.8%)
cT3	15 (10.5%)
Clinical *N* classification	
cN0	51 (35.6%)
cN1	83 (58.1%)
cN2	9 (6.3%)
IHC-based subtype	
Luminal	8 (5.6%)
Her-2+	74 (51.7%)
TNBC	61 (42.7%)
Ki-67	
<20%	19 (13.3%)
≥20%	124 (86.7%)
Chemotherapy regimen	
Anthracycline +Taxane	75 (52.4%)
Taxane+Carboplatin	44 (30.8%)
Taxane only	22 (15.4%)
Anthracycline-based only	2 (1.4%)
Targeted therapy	
trastuzumab	16 (21.6%)
trastuzumab+pertuzumab	10 (13.5%)
no targeted therapy	48 (64.9%)
Chemotherapy schedule	
3-week	98 (68.5%)
weekly	45 (31.5%)
Duration of chemotherapy	
<12 weeks	125 (87.4%)
12–16 weeks	18 (12.6%)
Adjuvant chemotherapy	
No	128 (89.5%)
Yes	15 (10.5%)
Surgery type of breast	
Partial mastectomy	7 (4.9%)
Total mastectomy	136 (95.1%)
Surgery type of axilla	
SLNB	18 (12.6%)
ALND	125 (87.4%)
Pathological results of breast	
No residual cancer	118 (82.5%)
DCIS	25 (17.5%)
Response of axilla	
No Response	78 (54.5%)
1–3 nodes Chemo-response	58 (40.6%)
>4 nodes Chemo-response	7 (4.9%)

HER-2+ human epidermal growth factor receptor 2 postive; TNBC, triple negative breast cancer; SLNB, sentinel lymph node biopsy; ALND, axillary lymph node dissection; DCIS, ductal carcinoma *in situ*.

### 3.2 Survival outcomes and prognostic analysis by molecular subtypes

**FIGURE 2:**
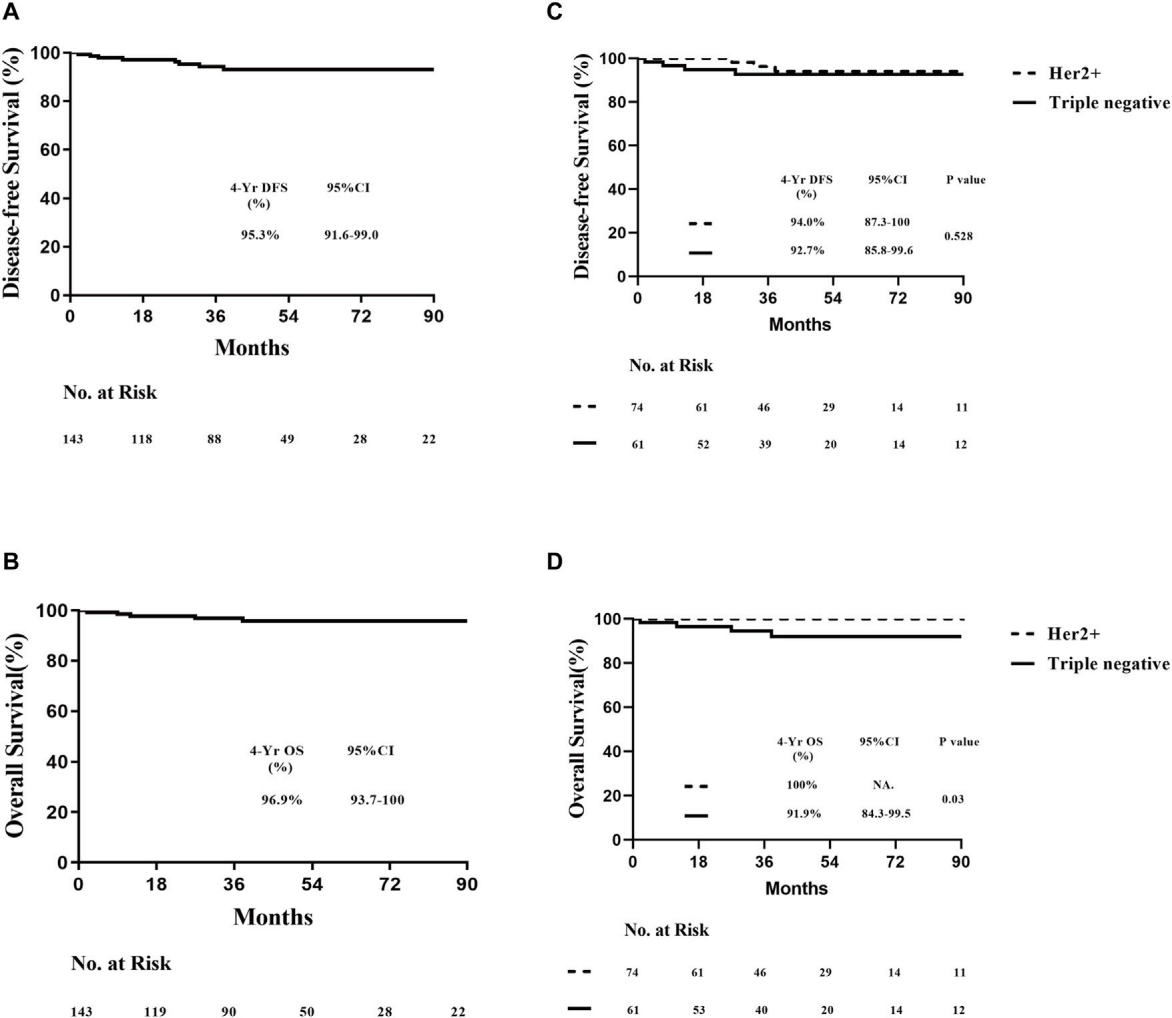
Prognosis of all patients who achieved pCR, including HER2-positive and triple-negative breast cancer. **(A)** DFS. **(B)** OS. **(C)** DFS of HER2-positive and triple-negative breast cancer across subtypes. **(D)** OS of HER2-positive and triple-negative breast cancer across subtypes. DFS, disease-free survival; and OS, overall survival.

**FIGURE 3:**
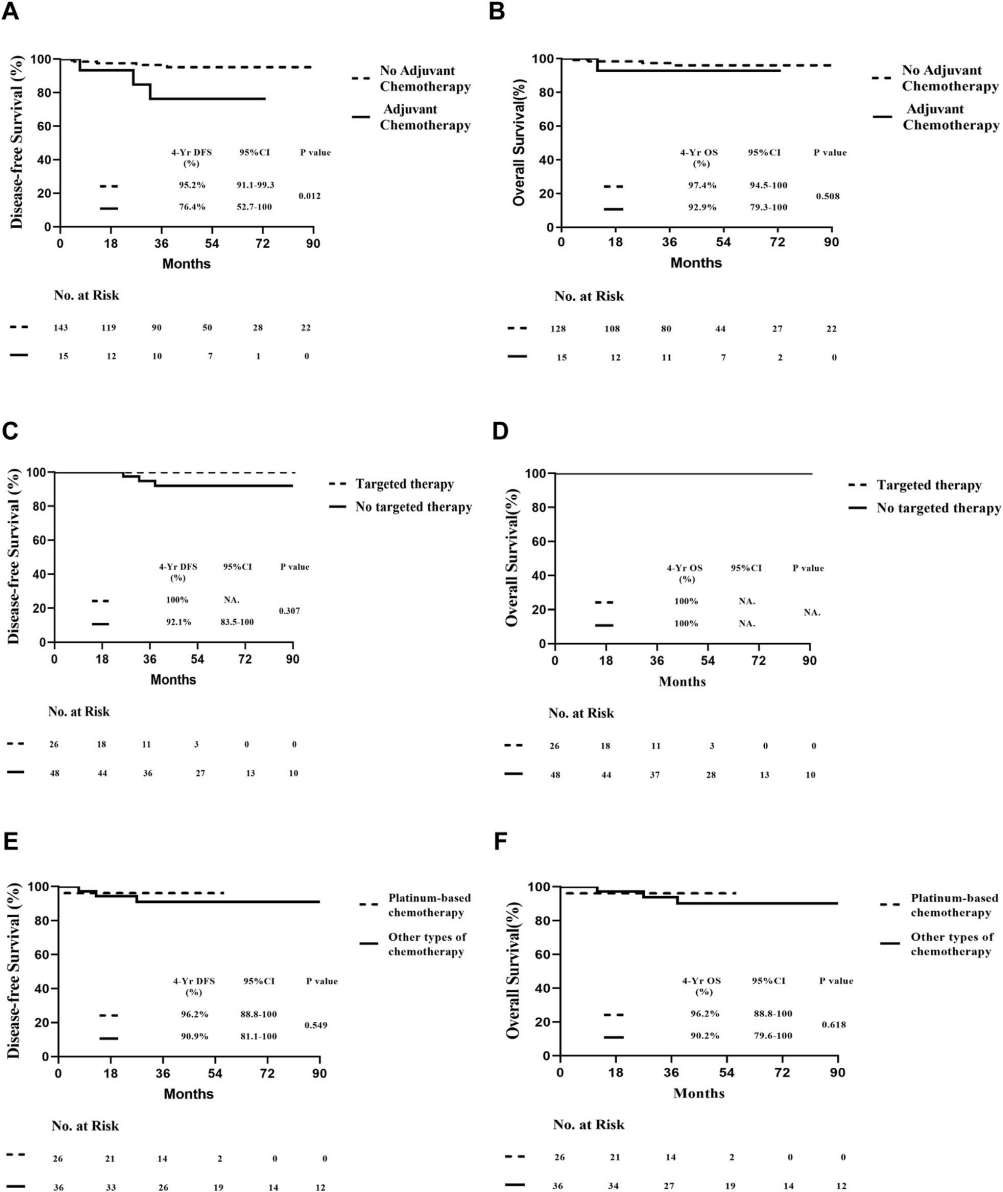
Prognosis of all patients who underwent pCR undergoing different therapies. **(A)** DFS of different adjuvant chemotherapy postoperatively. **(B)** OS of different adjuvant chemotherapy postoperatively. **(C)** DFS of HER2-positive pCR patients with or without target therapy. **(D)** OS of HER2-positive patients who achieved pCR with or without target therapy. **(E)** DFS of patients with triple-negative breast cancer who achieved pCR who did and did not undergo platinum drug therapy. **(F)** OS of patients with triple-negative breast cancer who achieved pCR, who did and did not undergo platinum drug therapy.

The median follow-up time for survival analysis was 47 months. Four-year overall DFS was 95.3% (95% CI, 91.6%–99.0%), and OS was 96.9% (95% CI, 93.7%–100%) in 143 patients. Among 74 HER2-positive and 61 TNBC patients, the 4-year DFS was 94.0% (95% CI, 87.3%–100%) and 92.7% (95% CI, 85.8%–99.6%), respectively, while the 4-year OS was 100% and 91.9% (95% CI, 84.3%–99.5%), respectively, with a statistically significant difference between both groups (*P* < 0.05; Figure 2). In the overall population, patients were divided into treatment and non-treatment groups according to whether adjuvant chemotherapy was given postoperatively. The 4-year DFS was 76.4% (95% CI, 52.7%–100%) and 95.2% (95% CI, 91.1%–99.3%) in the treatment and non-treatment groups, respectively, demonstrating statistically significant differences (*P* < 0.05). Meanwhile, the 4-year OS in the two groups was 92.9% (95% CI, 79.3%–100%) and 97.4% (95% CI, 94.5%–100%), respectively, demonstrating no statistically significant difference (Figures 3A,B). For patients who achieved pCR, DFS and OS were not inferior to those who received adjuvant therapy, with even superior DFS observed in patients who did not undergo adjuvant treatment. Second, we analysed whether HER2+ or TNBC affected the prognosis of patients who achieved pCR according to the treatment modalities of the different subgroups of HER2+ or TNBC. Patients with HER2+ breast cancer were divided into targeted therapy and non-targeted therapy groups, according to whether targeted therapy was applied during neoadjuvant chemotherapy. DFS at 4 years was 100% and 92.1% (95% CI, 83.5%–100%) in the targeted and no targeted treatment groups, respectively. OS at 4 years was 100% in both groups (Figures 3C,D). Similarly, according to the use of platinum agents for TNBC, we divided patients into platinum-based and non-platinum-based treatment groups, and the 4-year DFS was 96.2% (95% CI, 88.8%–100%) and 90.9% (95% CI, 81.1%–100%). OS was 96.2% (95% CI, 88.8%–100%) and 90.2% (95% CI, 79.6%–100%) in both groups, respectively (Figures 3E,F). In HER2-positive breast cancer, the administration of targeted therapy did not impact DFS or OS. Similarly, in TNBC, no significant differences in DFS or OS were observed between patients who received platinum-based therapy and those who did not. Among all analysed patients, a total of eight endpoint events related to recurrence or death were observed. Of these, three patients experienced local recurrence, while five developed distant metastases. Further stratification by molecular subtypes revealed distinct patterns of recurrence. In the HER2-positive subgroup, all three recurrence events were localized to the primary site. In contrast, the triple-negative breast cancer (TNBC) subgroup exhibited a more aggressive metastatic pattern, with all four recurrence events presenting as distant metastases. Specifically, the metastatic sites in TNBC patients included 2 cases of brain metastasis, 1 case of liver metastasis, and 1 case of lung metastasis (Table 2). Thus, it can be concluded that “de-escalation” therapy is feasible in such patients and may reduce complications associated with adjuvant therapy.

**TABLE 2:** Events observed for the primary end Point of disease-free survival.

Event (breast cancer subtypes)	Patients (N = 143) No. (%)	Time to Event Months
Any recurrence or death	8 (5.6%)	
Local or regional recurrence		
Ipsilateral axilla, (HER2-positive)	1 (0.7%)	32
Ipsilateral breast, (HER2-positive)	2 (1.4%)	13, 26
Distant recurrence		
Brain, (Triple negative and HR positive)	3 (2.1%)	2, 5, 7
Liver, (Triple negative)	1 (0.7%)	27
Lung, (Triple negative)	1 (0.7%)	38

HER-2, Human epidermal growth factor receptor 2; HR, hormone receptor.

### 3.3 Impact of adjuvant therapy on DFS and OS in pCR patients

**FIGURE 4:**
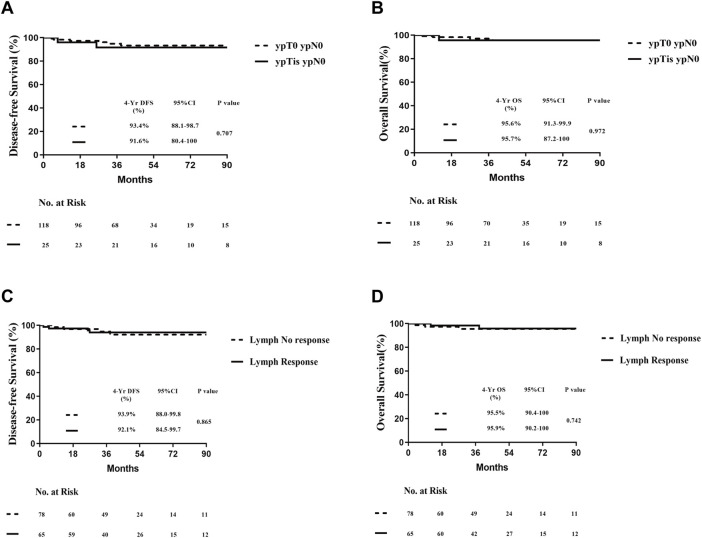
Prognosis of all patients with pCR according to the treatment pathological response postoperatively. **(A)** DFS of different types of breast residual pathology postoperatively. **(B)** OS of different types of breast residual pathology postoperatively. **(C)** DFS of different types of lymph-nodes residual pathology postoperatively. **(D)** OS of different types of lymph-nodes residual pathology postoperatively.

Subsequently, we further investigated whether variations in pathological treatment responses influence the prognosis of patients achieving pathological complete response (pCR) postoperatively. Breast pathology was evaluated based on the presence or absence of residual intraductal carcinoma. No significant differences in disease-free survival (DFS; log-rank test, *P* = 0.707) or overall survival (OS; log-rank test, *P* = 0.972) were observed between the two groups (Figures 4A,B). Similarly, differences in pathological lymph node response did not significantly impact DFS or OS following neoadjuvant therapy in patients with pCR (Figures 4C,D). These findings suggest that the presence or absence of ductal carcinoma *in situ* (DCIS) and lymph node response after neoadjuvant therapy do not alter the conclusion regarding the feasibility of “de-escalation” therapy in this patient population.

### 3.4 Pathological response and feasibility of de-escalation therapy

Univariate Cox regression analysis demonstrated that patients who underwent postoperative adjuvant therapy exhibited a significantly elevated risk of recurrence compared to those who did not receive adjuvant therapy (HR, 5.14; 95% CI, 1.2–21.5; *P* = 0.02). In contrast, other clinical factors, such as age (<50 vs ≥50 years), breast cancer subtype, duration of chemotherapy (<12 vs 12–16 weeks), pretreatment tumor size, and pretreatment lymph node status, showed no significant association with recurrence risk. To further validate these findings, significant risk factors identified in the univariate analysis were incorporated into a multivariate Cox proportional hazards model. The results consistently indicated that postoperative adjuvant chemotherapy was an independent predictor of prognosis (*P* = 0.02; Table 3). These findings highlight the critical role of postoperative adjuvant therapy in modulating recurrence risk, even among patients who achieved pathological complete response (pCR).

**TABLE 3:** Cox proportional hazard model of predictors of cancer recurrence in patients who achieved pCR.

Predictors		Univariate analysis			Multivariate analysis		
		Harzard ratio	95% CI	*P* Value	Harzard ratio	95% CI	*P* Value
Age at diagnosis (years)	<50	Ref					
	≥50	0.89	0.20–3.70	0.87			
Clinical stage	II	Ref					
	III	1.16	0.10–9.40	0.89			
Clinical T classifification	cT1	Ref					
	cT2	1.89	0.23–15.64	0.56			
	cT3	2.35	0.15–37.55	0.55			
Clinical N classifification	cN0	Ref					
	cN1	1.72	0.33–8.89	0.52			
	cN2	2.75	0.25–30.30	0.41			
Her-2 status	Negative	Ref					
	Postive	0.42	0.10–2.30	0.41			
HR status	Negative	Ref					
	Postive	1.14	0.20–5.70	0.87			
Adjuvant chemotherapy	No	Ref			Ref		
	Yes	5.14	1.20–21.50	**0.02**	6.27	1.40–28.00	**0.02**
Schedule	3-weekly	Ref					
	Weekly	0.03	0–12.20	0.25			
Duration of chemotherapy	<12 weeks	Ref					
	12–16 weeks	0.04	0–140.00	0.44			
Ki-67	<20	Ref					
	≥20	1.32	0.20–10.80	0.80			

Abbreviations: Her-2, Human epidermal growth factor receptor 2; HR,hormone receptor.

## 4 Discussion

Numerous meta-analyses have confirmed that high-risk breast cancer patients (TNBC or HER2+) who achieve pCR following neoadjuvant chemotherapy exhibit favorable prognoses (Cortazar et al., 2014; von Minckwitz et al., 2012; LeVasseur et al., 2020). However, as observed in our central analysis, several studies have identified risk factors influencing prognosis in pCR patients, including pretreatment tumor size, lymph node metastasis, clinical stage, molecular subtype, and treatment modalities (Asaoka et al., 2019; van Mackelenbergh et al., 2023; Huang et al., 2021; Chaudry et al., 2015). This study focused on high-risk cII–cIIIA HER2+ or TNBC patients with relatively mild clinical stages who achieved pCR after short-term neoadjuvant chemotherapy (≤16 weeks), often combined with targeted therapy. Over a 4-year follow-up, the disease-free survival (DFS) and overall survival (OS) rates were 95.3% and 96.9%, respectively. Specifically, HER2+ patients demonstrated a DFS of 94.0% and an OS of 100%, while TNBC patients showed a DFS of 92.7% and an OS of 91.9%. These outcomes are comparable to, or even slightly superior to, those reported in previous neoadjuvant prospective studies for these subtypes. Notably, the treatment duration in this study was significantly shorter than in prior studies, suggesting that neoadjuvant chemotherapy does not compromise prognosis while offering better tolerance, fewer side effects, and reduced medical resource utilization.

Further analysis revealed that adjuvant chemotherapy did not improve prognosis in pCR patients. In fact, patients receiving adjuvant therapy had worse outcomes, with a 4-year DFS of 76.4% compared to 95.2% in those who did not receive adjuvant therapy (*P* = 0.012). This discrepancy may stem from the fact that patients receiving adjuvant therapy tended to have more advanced clinical stages at baseline or were more likely to complete conventional treatment cycles based on physician or patient preferences. Additionally, we evaluated the impact of treatment regimens (e.g., platinum-based therapy in TNBC or targeted therapy in HER2+ patients) on prognosis and found no significant differences in outcomes among pCR patients. For instance, HER2+ patients receiving targeted therapy achieved a 4-year DFS of 100%, with no recurrence observed. These findings align with a previous meta-analysis, which concluded that adjuvant chemotherapy post-pCR had minimal impact on further improving prognosis (non-adjuvant vs adjuvant 5-year EFS: 88% vs 86%, P = 0.60) (Spring et al., 2020). Emerging evidence suggests that chemotherapy may induce tumor immunosuppression by inhibiting immune cells such as T cells and dendritic cells, thereby reshaping the tumor microenvironment (Sharma et al., 2024). Additionally, research published in Nature identified a protein, RHOJ, that promotes DNA damage repair in cancer cells undergoing epithelial-mesenchymal transition (EMT), enabling resistance to chemotherapy (Debaugnies et al., 2023). These findings underscore the need for further investigation into the role of adjuvant therapy following neoadjuvant chemotherapy.

The analysis demonstrates that operable cII–cIIIA HER2+ or TNBC patients who achieve pCR after short-term neoadjuvant chemotherapy (with or without targeted therapy) exhibit excellent prognosis, comparable to pCR patients in prospective neoadjuvant studies with conventional treatment durations and even approaching the outcomes of low-risk breast cancer patients in the SEER database. This parallels findings from the adjuvant anti-HER2+ APT study, where early-stage HER2+ breast cancer patients achieved a 4-year IDFS of 98.7% and RFS of 99.2% with low-intensity chemotherapy combined with targeted therapy (Tolaney et al., 2015). These results suggest limited room for further prognostic improvement through intensified treatment, often at high cost. In this study, adjuvant therapy did not enhance prognosis in pCR patients, highlighting the potential to revise traditional neoadjuvant treatment paradigms. Given that neoadjuvant regimens are typically derived from adjuvant regimens and do not impact overall survival (Early Breast Cancer Trialists' Collaborative Group, 2018), there is growing interest in “tailored therapy” approaches. Recent studies have explored “de-escalation therapy” based on treatment response during neoadjuvant therapy to address overtreatment (Gupta et al., 2022; Leon-Ferre et al., 2021; Miglietta et al., 2021). The favorable prognosis observed in our cohort supports the feasibility of neoadjuvant de-escalation strategies and identifies a suitable patient population for such approaches. However, further prospective studies, including the ongoing CompassHER2 trial, are needed to provide robust clinical evidence and refine current treatment paradigms (Werutsky and Rosa, 2020).

This study has several limitations. As a retrospective analysis, residual confounding cannot be entirely ruled out, though we mitigated this risk by adjusting for clinically relevant covariates. The single-center, small-sample design may limit the generalizability of our findings, and the relatively short follow-up duration necessitates longer-term data to enhance clinical applicability. Additionally, while prior studies from our center identified treatment regimens as prognostic factors in pCR patients (Huang et al., 2021), this study did not reflect the impact of targeted therapy in HER2+ breast cancer, possibly due to the relatively mild pretreatment stages of the included patients. Conversely, the poorer prognosis observed in patients receiving adjuvant chemotherapy suggests that advanced clinical stages may require more intensive treatment regimens, consistent with our previous findings. Future studies with larger sample sizes and multi-center prospective designs are warranted to validate these conclusions and provide more reliable evidence.

## 5 Conclusion

In conclusion, patients with operable cII–cIIIA breast cancer who achieve pCR following short-term neoadjuvant chemotherapy (combined with targeted therapy)—representing those with relatively early clinical stages and high sensitivity to neoadjuvant treatment—exhibit an excellent prognosis. Based on the findings of our study, chemotherapy “de-escalation” appears to be a viable strategy for this population. This approach aligns with the concept of “tailored” neoadjuvant therapy, where treatment intensity is stratified and beneficiaries are identified based on therapeutic response. Our results provide robust evidence to support the implementation of neoadjuvant therapy in a more personalized manner. Moving forward, conducting targeted studies, including the identification of biomarkers predictive of treatment sensitivity, will be crucial for advancing precision medicine in cancer treatment and optimizing therapeutic outcomes.

## Data Availability

The original contributions presented in the study are included in the article/Supplementary Material, further inquiries can be directed to the corresponding authors.
