# Supplementary material for: Screening and exploration of neoadjuvant “de-escalation” therapy for early breast cancer
Source: Front Pharmacol. 2025 Mar 25;16:1574665. doi: 10.3389/fphar.2025.1574665 (PMC11975863; doi:10.3389/fphar.2025.1574665)
Supplement: Supplementary file 1 [file Table1.docx]

**Supplement Table S1. The Neoadjuvant Clinical Trials of Her2+ and TNBC**

|  |  |  |  |  |  |  |  |  |
| --- | --- | --- | --- | --- | --- | --- | --- | --- |
| **Clinical Trials** | | **Entry Population** | **Clinical Staging** | **Treatment options** | **Duration of neoadjuvant** | **PCR Rate** | **PCR Prognosis** | |
|  |  |  |  |  |  |  | **EFS** | **OS** |
| **Her2+** |  |  |  |  |  |  |  |  |
|  | **TRAIN-2** | 438 | II - III | TCbHP | 9*q3w | 68.00% | **3y- 94.1%** | **_ _** |
|  |  |  |  | FECHP-TCbHP | 3*q3w - 6*q3w | 67.00% |  |  |
|  | **KRISTINE** | 444 | II - IIIc | TCbHP | 6*q3w | 55.70% | **3y- 97.2%** | **_ _** |
|  |  |  |  | T-DM1+P |  | 44.40% |  |  |
|  | **CALGB 40601** | 305 | II - III | THL | 16*qw | 56.00% | **7y- 89%** | **7y- 95%** |
|  |  |  |  | TH |  | 46.00% |  |  |
|  |  |  |  | TL |  | 32.00% |  |  |
|  | **NeoALTTO** | 455 | II - III | L-TL | 6*qw - 12*qw | 20.00% | **6y- 77%** | **6y- 89%** |
|  |  |  |  | H-TH |  | 27.60% |  |  |
|  |  |  |  | LH-THL |  | 46.80% |  |  |
|  | **HannaH** | 523 | I - IIIc | TH-FECH  (ivgtt vs ih) | 4*q3w - 4*q3w | 34.20% | **6y- 82%** | **_ _** |
|  |  |  |  |  |  | 39.20% |  |  |
| **TN** |  |  |  |  |  |  |  |  |
|  | **BrightNess** | 634 | II - III | PCbV-AC | 4*q3w - 4*q2w | 53.00% | **4y- 89%** | **_ _** |
|  |  |  |  | PCb-AC |  | 58.00% |  |  |
|  |  |  |  | P-AC |  | 31.00% |  |  |
|  | **KEYNOTE-522** | 1174 | II - III | PCbPem-ACPem | 12*qw - 4*q3w | 64.80% | **3y- 94.2%** | **_ _** |
|  |  |  |  | PCb-AC |  | 51.20% | **3y- 92.5%** |  |
|  | **IMpassion031** | 333 | II - III | nabPAtezo-ECAtezo | 12*qw - 4*q2w | 58.00% | **2y- 92.6%** | **_ _** |
|  |  |  |  | nabP-EC |  | 41.00% | **2y- 91.3%** |  |
|  | **ARTemis** | 241 | II - III | T-FEC | 3*q3w - 3*q3w | 31.00% | **3y- 90%** | **_ _** |
|  |  |  |  | TBev-FECBev |  | 45.00% |  |  |

HER-2+ human epidermal growth factor receptor 2 postive; TNBC triple negative breast cancer.
